# Exoribonuclease-Resistant RNAs Exist within both Coding and Noncoding Subgenomic RNAs

**DOI:** 10.1128/mBio.02461-18

**Published:** 2018-12-18

**Authors:** Anna-Lena Steckelberg, Quentin Vicens, Jeffrey S. Kieft

**Affiliations:** a Department of Biochemistry and Molecular Genetics and RNA BioScience Initiative, University of Colorado Denver School of Medicine, Aurora, Colorado, USA; University of Melbourne; UW-Madison; Iowa State University

**Keywords:** *Infernal*, RNA structure, exoribonuclease resistance, plant viruses, viral RNA

## Abstract

Many viruses produce protein-coding and noncoding subgenomic RNAs (sgRNAs) that are critical for infection. A recently discovered pathway for viral sgRNA production uses exoribonuclease-resistant RNAs (xrRNAs), discrete folded RNA elements that block the processive exoribonucleolytic degradation of RNA. xrRNAs are widespread in animal-infecting flaviviruses but had been found only in three members of the plant virus genus *Dianthovirus*. Also, xrRNAs had been found only in the 3′ untranslated regions (3′UTRs) of viral RNAs, where they produce noncoding sgRNAs. The degree to which xrRNA elements exist in other viruses, the conservation of their ring-like fold, and the ability of xrRNAs to operate in diverse contexts were unknown. Using computational tools and biochemical assays, we discovered xrRNA elements pervading two large families of plant-infecting RNA viruses, demonstrating their importance and widespread utility. Comparison of the sequences and functional requirements suggests that all adopt the characteristic ring-like fold. Unexpectedly, many of these newly discovered xrRNAs are located in intergenic regions rather than 3´UTRs, and some are associated with the 5′ ends of subgenomic RNAs that encode viral proteins. This suggests that xrRNAs are involved in the production of both coding and noncoding subgenomic RNAs and can operate as part of broader mechanisms to regulate RNA levels and protein expression. These discoveries expand the potential roles for xrRNAs and suggest that xrRNAs may represent a more general strategy for RNA maturation and maintenance than previously known.

## Abstract (precis)

During infection, viruses often produce subgenomic RNAs (sgRNAs) that either serve as the template for protein synthesis or act as “riboregulators” that interact with and influence the viral and cellular machinery. Recently, a mechanism for producing sgRNAs was found that depends on the presence of specifically structured RNA elements (xrRNAs). However, the degree to which this mechanism is used, where the elements are found, their structural diversity, and what types of sgRNAs are produced by this pathway were unclear. This article describes the discovery of these structured RNA elements in two large families of plant viruses and shows that they are used to produce both protein-coding sgRNAs and “riboregulatory” RNAs. These discoveries provide evidence that xrRNA-based RNA maturation pathways may be more widespread than previously anticipated and that they are involved in producing a variety of RNAs of diverse functions.

## INTRODUCTION

**FIG 1:**
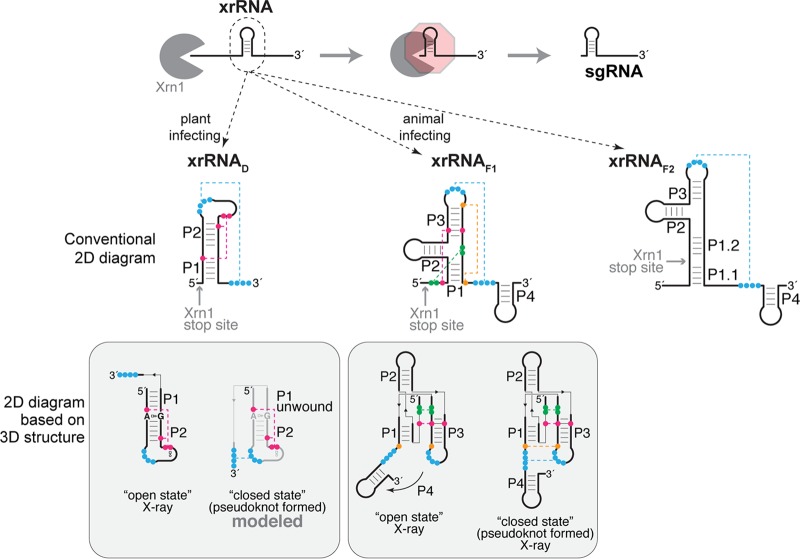
An expanding repertoire of structured RNAs for blocking exoribonuclease degradation. (Top) xrRNAs adopt a three-dimensional structure that blocks the progression of 5′-to-3′ exoribonucleases such as Xrn1 (gray). In the case of flaviviruses and dianthoviruses, xrRNAs are in the 3′UTR, resulting in accumulating noncoding sgRNAs. (Middle) Secondary structure diagrams of the two classes of xrRNAs from flaviviruses (xrRNA_F1_ and xrRNA_F2_) (15, 22, 23) and of xrRNA_D_ from dianthoviruses (26). Secondary structure features are labeled, and nucleotides involved in tertiary interactions are shown in colors connected by dashed lines (pseudoknot shown in blue). Experimentally determined Xrn1 stop sites are indicated. (Bottom) The boxes below each secondary structure contain diagrams reflecting the currently available three-dimensional structures (24–26). The A8-G33 pair is highlighted in the open state of the Sweet clover necrotic mosaic virus (SCNMV) xrRNA (far left).

Single-stranded positive-sense RNA viruses include pathogens that infect a wide range of animal and plant hosts, with significant human health, agricultural, and economic impact. During infection, these viruses must generate many copies of their full-length genomic RNA, but many also produce one or more infection-critical subgenomic RNA species (sgRNA). sgRNAs can have different functions: they can encode and serve as the template for production of specific viral proteins or act as noncoding “riboregulators” that interact with and influence the cellular and viral machinery to benefit viral infection (1–6). Most viral sgRNAs are thought to be produced directly through transcription from internal subgenomic promoters or by premature termination during negative-strand synthesis (4). However, recent discoveries showed that some noncoding viral sgRNAs result from incomplete degradation of the genomic RNA in a pathway depending on discrete, compact RNA structures that block the progression of 5′-to-3´ exoribonucleases (Fig. 1) (7–15). The full extent of this phenomenon and the diversity of RNA structures that can provide this ability are unknown, and to date all have been associated with noncoding RNA production.

Exoribonuclease-resistant RNA (xrRNA) elements were first identified in mosquito-borne flaviviruses (e.g., dengue virus, Zika virus, West Nile virus), where they protect the genome’s 3′ untranslated region (3′UTR) from degradation (8). The resultant decay intermediates accumulate and comprise biologically active viral noncoding sgRNAs (Fig. 1) (8, 9, 12, 16–21). xrRNAs are useful and important elements, as they are broadly found within the 3′UTRs of flaviviruses, including those that are tick-borne; are specific to insects; or have no known vector (15, 22, 23). Comparison and characterization of these diverse flaviviral xrRNA (xrRNA_F_) sequences revealed two classes; class I (xrRNA_F1_) is exemplified by mosquito-borne flaviviruses, whereas class II (xrRNA_F2_) is found in diverse flaviviruses (23). Extensive functional and high-resolution structural studies of xrRNA_F1_ have shown that function is conferred by a specific three-dimensional (3D) fold containing an interwoven pseudoknot stabilized by conserved secondary and tertiary interactions; this creates an unusual ring-like conformation that protectively wraps around the 5′ end of the RNA structure (24, 25). Although aligned xrRNA_F2_ sequences show conserved patterns, their 3D structures are unknown (23).

As with xrRNA_F2_, the structures of recently reported xrRNAs from most other viral clades remain unsolved (7, 13), with the exception of those found in a small genus of plant-infecting RNA viruses. Specifically, we recently characterized the structure and function of xrRNAs from the 3′UTRs of dianthoviruses, which are positive-sense RNA viruses in the *Tombusviridae* family; similarly to xrRNA_F_, dianthoviral xrRNAs (xrRNA_D_) function to produce a noncoding RNA derived from the viral 3′UTR (10, 26). xrRNA_D_ also rely on a pseudoknot that forms a protective ring-like structure (26), but they have very different sequences and secondary structures from those of xrRNA_F1_, and the ring is formed by a different set of interactions (Fig. 1). The xrRNA_D_ crystal structure is in an “open” conformation that likely represents a folding intermediate whose presence is necessary before the pseudoknot forms (26) (Fig. 1). Thus, we still do not know the full repertoire of secondary and tertiary interactions required to form and stabilize the exoribonuclease-resistant pseudoknot state of xrRNA_D_. In addition, because only 3 examples are known, the lack of diverse xrRNA_D_ sequences prevents conclusions about the role, prevalence, and structural diversity of this fold.

Because xrRNA_F_ elements pervade the flaviviruses with associated sequence and structural diversity characteristics, it was puzzling that xrRNA_D_ had been identified only in the three closely related members of the *Dianthovirus* genus. This observation raised the issue of whether xrRNAs similar to xrRNA_D_ are more widespread and diverse than currently known; if so, it would indicate that they represent a more general and perhaps more important way to produce or protect viral RNAs than is currently recognized. This is true in a broader sense as well; the issue of how widespread xrRNAs are across biology remains open.

To begin to address both the specific issue of the presence of xrRNA_D_ in other viruses and the more general issues about xrRNA diversity and distribution, we used a bioinformatic approach to search for more xrRNA_D_ in a variety of plant viruses. We identified over 40 putative new xrRNA_D_-like elements in viruses belonging to the economically important *Tombusviridae* and *Luteoviridae* families. *In vitro* assays showed that these elements are indeed resistant to Xrn1, and analysis of these new xrRNAs revealed both conservation and variability. Surprisingly, we found many of these xrRNAs in intergenic regions of the viral genomic RNA, where they can be involved in the generation or maintenance of sgRNA species with protein-coding potential; hence, xrRNAs are not limited to noncoding RNA generation. These discoveries provide evidence that xrRNA-based RNA maturation pathways may be more widespread than previously anticipated and are involved in producing a variety of RNAs of diverse function.

## RESULTS AND DISCUSSION

To search for new xrRNA_D_-like elements, we used the *Infernal* software (S. R. Eddy laboratory), which enables screening of massive data sets of DNA sequences for conserved RNA secondary structure patterns with poor sequence conservation (27). Because the *Dianthovirus* genus has only three members (Red clover necrotic mosaic virus [RCNMV], Sweet clover necrotic mosaic virus [SCNMV], and Carnation ringspot virus [CRSV]) (26), we expanded our search to other plant-infecting positive-sense RNA viruses. The initial search within a library of viral reference genomes (see Materials and Methods) identified two potential sequences in the *Luteoviridae* corresponding to the poleroviruses wheat leaf yellowing-associated virus isolate JN-U3 (GenBank accession no. NC_035451; *Infernal* E value = 0.00025, score = 44.3) and sugarcane yellow leaf virus (GenBank accession no. NC_000874; *Infernal* E value = 6.5, score = 24.2). With these sequences added to the alignment, subsequent searches identified >40 candidates within the public repository of all available sequences for *Tombusviridae* and *Luteoviridae*, demonstrating how powerful this tool is for computationally identifying putative functional elements in viral RNAs (28).

**FIG 2:**
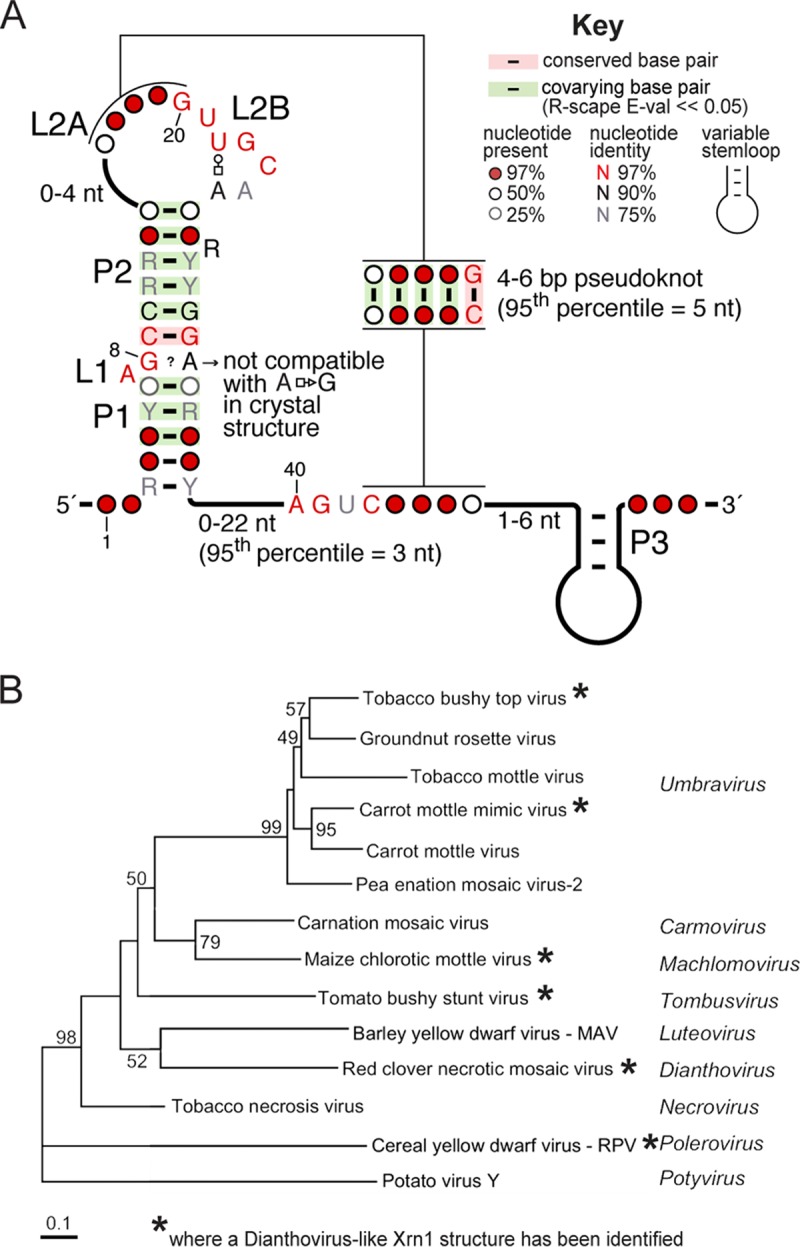
Widespread occurrence of Xrn1-resistant RNAs among plant viruses. (A) Consensus sequence and secondary structure of xrRNA_D_ based on a comparative sequence alignment of 47 sequences of viruses belonging to the *Tombusviridae* and *Luteoviridae* families (shown in Fig. S1 in the supplemental material). Y = pyrimidine; R = purine. Non-Watson-Crick base pairs are shown using the Leontis-Westhof nomenclature (49). The numbering is that of the crystal structure of the SCNMV xrRNA (26). (B) Phylogenetic relationship between various plant viruses, based on the RNA-dependent RNA polymerase amino acid sequence (31). The viruses and corresponding genera in which we identified xrRNA_D_ structures are marked by a star. Numbers at the nodes refer to bootstrap values as percentages obtained from 2,000 replications, shown only for branches supported by more than 40% of the data. Branch lengths are proportional to the number of changes. Further analysis will likely reveal xrRNA_D_ elements in more of these viruses with additional sequence and structural variation.

A close inspection of all putative xrRNA_D_-like elements revealed that their predicted secondary structures contain the same elements as were found in the known xrRNA_D_. Specifically, the assertion of formation of helices P1 and P2 and a predicted pseudoknot is supported by covariation data, which reveal little sequence conservation (R-scape [29] E values for the 12 covarying base pairs in the stems and the pseudoknot are within 3.10^−4^ to 8.10^−13^ (95th percentile = 1.10^−12^); Fig. 2A). Notably, our search criteria did not contain the pseudoknot interaction; thus, the fact that all putative xrRNA_D_ sequences have the ability to form this functionally important element serves as internal cross-validation. L1 and L2B are >97% conserved in sequence, consistent with their role in creating a specific folded motif that promotes pseudoknot formation (26). Also, two of the three nucleotides immediately upstream of the 3′ side of the pseudoknot are >97% conserved, but their role is not obvious from the crystal structure of the open state. Likewise, the non-Watson-Crick A8-G33 base pair identified in the crystal structure (Fig. 1) cannot be reconciled with the predominant presence of G at position 8 and G/A at position 33 in all the other sequences. These observations support the previous assertion that the crystallized open state represents a folding intermediate of xrRNA_D_ and that structural adjustments and additional interactions are present in the “closed” pseudoknot state.

10.1128/mBio.02461-18.1 FIG S1 Sequence alignment of computationally identified viruses comprising a Dianthovirus-like xrRNA (Stockholm format). Download FIG S1, TIF file, 12.6 MB. Copyright © 2018 Steckelberg et al. 2018 Steckelberg et al. This content is distributed under the terms of the Creative Commons Attribution 4.0 International license.

Viruses in which we found putative novel xrRNAs include members of the *Tombusviridae* and *Luteoviridae* families. In the *Tombusviridae*, xrRNAs were found in the *Machlomovirus* and *Umbravirus* genera. In the *Luteoviridae* family, members of the *Polerovirus* and *Enamovirus* genera contain putative xrRNAs. We did not find putative xrRNAs in the *Luteovirus* genus despite its close relationship to the *Dianthovirus* or in the *Sobemovirus* genus, which is closely related to *Polerovirus.* It is possible that these viruses do not have xrRNAs or that they may have xrRNAs that are more divergent in sequence and secondary structure and thus would not be identified with our search criteria. We chose to remain conservative with respect to this search; future work may identify new elements in these viruses as well.

**FIG 3:**
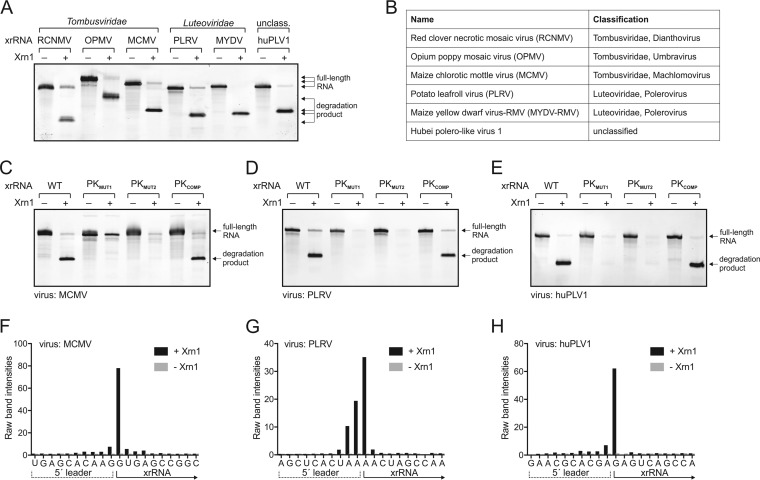
Biochemical characterization of representative plant virus xrRNA_D_ elements. (A) *In vitro* Xrn1 resistance assay of xrRNA_D_ from various plant RNA viruses (Table 1). The xrRNA from RCNMV was included as a positive control. Arrows indicate the size of full-length RNAs and Xrn1-resistant degradation products. (B) Classification of viruses used in the experiments represented in panel A (Table 1). (C to E) *In vitro* Xrn1 resistance assay of wild-type (WT) and pseudoknot (PK) mutant versions of MCMV (C), PLRV (D), and HuPLV1 (E) xrRNAs. (F to H) Reverse transcription (RT) mapping of the Xrn1 stop site. Data represent distributions of RT products of Xrn1-resistant fragments of MCMV (F), PLRV (G), and HuPLV1 (H) degradation fragments. Experimentally validated stop sites are indicated on the secondary structure diagrams for all tested xrRNA_D_ shown in Fig. S2.

To experimentally determine if the computationally identified elements were authentic xrRNAs, we tested representative sequences from viruses of both families using our established *in vitro* Xrn1 resistance assay (11). Specifically, *in vitro*-transcribed and purified RNA sequences from opium poppy mosaic virus (OPMV), Maize chlorotic mottle virus (MCMV), Potato leafroll virus (PLRV), Maize yellow dwarf virus-RMV (MYDV-RMV), and Hubei polero-like virus 1 (HuPLV1) were challenged with recombinant Xrn1. All RNAs stopped Xrn1 degradation similarly to positive-control RCNMV xrRNA_D_ (Fig. 3A and B), demonstrating that they are authentic xrRNAs that do not require additional *trans*-acting proteins for function. Moreover, mutations to disrupt the putative pseudoknot in the MCMV, PLRV, and HuPLV1 xrRNAs abolished Xrn1 resistance, while compensatory mutations that restore pseudoknot base pairing rescued the activity (Fig. 3C to E), verifying the functional importance of the pseudoknot in all of these examples. In addition, the mapped Xrn1 stop site is at the base of P1 in all newly identified xrRNAs, matching the xrRNA_D_ stop site (Fig. 3F to H; see also Fig. S2 in the supplemental material) (26). Overall, the conserved secondary structure (Fig. 2A), the location of the exoribonuclease stop site, and the strict dependence on the pseudoknot for Xrn1 resistance suggest that these newly identified and tested xrRNAs use molecular folds and mechanisms similar to those seen with xrRNA_D_. By extension, the same is very likely true of the larger set that we computationally identified; thus, we classify them as members of the xrRNA_D_ class of exonuclease-resistant RNA structures.

10.1128/mBio.02461-18.2 FIG S2 Predicted secondary structure diagrams for biochemically tested xrRNA_D_. Results of RT mapping of Xrn1 halt sites are shown for OPMV and MYDV-RMV. RCNMV data are from a previously published article (A. L. Steckelberg, B. M. Akiyama, D. A. Costantino, T. L. Sit, et al., PNAS **115**:6404–6409, 2018). Download FIG S2, JPG file, 0.3 MB. Copyright © 2018 Steckelberg et al. 2018 Steckelberg et al. This content is distributed under the terms of the Creative Commons Attribution 4.0 International license.

Although the newly identified xrRNA_D_ elements share many features, there are notable structural differences in a subset of xrRNAs found in the *Tombusviridae* family (RCNMV, SCNMV, CRSV, OPMV, and MCMV). Specifically, these xrRNA_D_ have a P3 stem-loop immediately downstream of the pseudoknot (Fig. 2A) (Table 1; see also Table S1 in the supplemental material). Although the presence of P3 was not recognized in the previous characterization of dianthovirus xrRNAs, the truncation analysis in that study showed that this part of the sequence is not required by xrRNA_D_ for Xrn1 resistance *in vitro* (26). Consistent with this, an analogous stem-loop (P4) found in xrRNA_F1_ is also dispensable *in vitro*; the crystal structure indicates that it may stabilize the pseudoknot through stacking interactions (Fig. 1) (24). Therefore, in xrRNA_D_, coaxial stacking of P3 on P1/P2 could similarly stabilize the RNA structure in the cell during infection, but it is not necessary in all contexts.

10.1128/mBio.02461-18.4 TABLE S1 Complete list of computationally identified viruses comprising a Dianthovirus-like xrRNA. Download Table S1, PDF file, 1.2 MB. Copyright © 2018 Steckelberg et al. 2018 Steckelberg et al. This content is distributed under the terms of the Creative Commons Attribution 4.0 International license.

**FIG 4:**
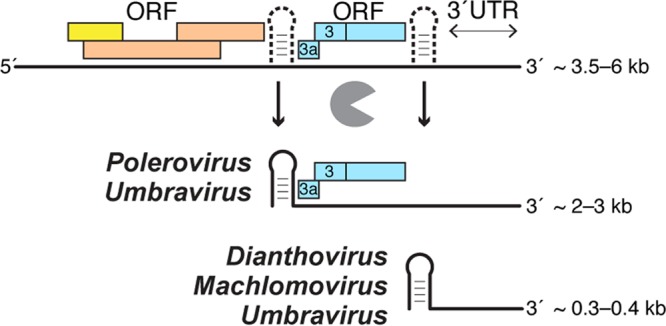
xrRNA_D_ can produce or protect both coding and noncoding sgRNAs. The presence of xrRNA_D_ in different contexts suggests an expanded role for these RNA elements. Full-length viral genomic RNA (top; colored boxes symbolize ORFs**)** can be processed by exoribonucleases that stop at xrRNAs (depicted as dashed structures) to yield both protein-coding sgRNAs (middle) and noncoding sgRNAs (bottom). Also, sgRNAs produced by subgenomic promoters could be “trimmed” or protected by xrRNAs (not shown). Only some umbraviruses (e.g., OPMV) possess two xrRNA_D_ elements. Note that the ORF organization characteristics are not identical in all of these viruses; thus, this depiction should be considered conceptual. Details of the genetic organization and xrRNA location can be found in Fig. S3.

All previously known xrRNAs lie upstream of noncoding RNAs and lead to the generation of sgRNAs that do not encode proteins; however, the location of the newly discovered xrRNAs reveals unexpected variation and thus potential new roles for xrRNAs in general (Fig. 4). Surprisingly, only two of the newly identified xrRNAs are in the 3′UTR of the viral genome (Table 1). In MCMV, the first nucleotide of the P1 helix matches the 5′ end of noncoding sgRNA2 (30); thus, as with the dianthoviruses, flaviviruses, and other xrRNAs, this new element probably blocks Xrn1 to generate noncoding sgRNAs derived from the 3′UTR. In contrast, for some members of the *Tombusviridae* family as well as for poleroviruses, xrRNA_D_ is located in an intergenic region 5 to 20 nucleotides upstream from the translation start site of open reading frame 3a (ORF3a) and ∼135 nucleotides from the start site of a readthrough protein encoded by ORF3 to ORF5 (ORF3–5) (our data suggest that ORF3a has not been annotated for all poleroviruses; see Table S1). ORF3a codes for protein P3a, which is essential for long-distance movement of the virus in plants (30). Translation of ORF3a initiates from sgRNA1 at a non-AUG codon (Table 1; see also Table S1) (30–32). The location of the xrRNA_D_ upstream of the ORF3a start site implies that these xrRNAs, rather than functioning in noncoding RNA production, might act to produce or maintain protein-coding RNAs (Fig. 4).

**TABLE 1:** Selected set of plant viruses possessing an xrRNA_D_^*a*^

Name	Abbreviation	Classification	GenBank accession no.	Length (nt)	Genomic location (nt)^*b*^	Genomic context
Red clover necrotic mosaic virus	RCNMV	*Tombusviridae*; *Dianthovirus*	NC_003756	3,890	3461–3504	3′ UTR
Sweet clover necrotic mosaic virus	SCNMV	*Tombusviridae*; *Dianthovirus*	NC_003806	3,876	3446–3489	3′ UTR
Maize chlorotic mottle virus (isolate KS1)	MCMV	*Tombusviridae*; *Machlomovirus*	NC_003627	4,437	4101–4143	3′ UTR
Opium poppy mosaic virus (isolate PHEL5235)	OPMV	*Tombusviridae*; *Umbravirus*	NC_027710	4,230	3585–3629	3′ UTR
Carrot mottle mimic umbravirus	CMoMV	*Tombusviridae*; *Umbravirus*	NC_001726	4,201	2664–2706	74 nt to AUG from ORF3
Chickpea chlorotic stunt virus	CpCSV	*Luteoviridae*; *Polerovirus*	NC_008249	5,900	3489–3534	11 nt to AUA from ORF3a; 129 nt to AUG from ORF3–5
Cowpea polerovirus 1 (isolate BE167)	CpPV1	*Luteoviridae*; *Polerovirus*	NC_034246	5,845	3380–3425	11 nt to CUG from ORF3a; 129 nt to AUG from ORF3–5
Cotton leafroll dwarf virus	CoLRDV	*Luteoviridae*; *Polerovirus*	NC_014545	5,866	3451–3499	13 nt to CUG from ORF3a; 131 nt to AUG from ORF3–5
Cereal yellow dwarf virus-RPV	CYDV-RPV	*Luteoviridae*; *Polerovirus*	NC_004751	5,723	3566–3622	14 nt to AUU from ORF3a; 132 nt to AUG from ORF3–5
Maize yellow dwarf virus-RMV (formerly BYDV)	MYDV-RMV	*Luteoviridae*; *Polerovirus*	NC_021484	5,612	3335–3384	14 nt to ACG from ORF3a; 132 nt to AUG from ORF3–5
Potato leafroll virus	PLRV	*Luteoviridae*; *Polerovirus*	NC_001747	5,987	3509–3557	18 nt to AUA from ORF3a; 136 nt to AUG from ORF3–5
Sugarcane yellow leaf virus	ScYLV	*Luteoviridae*; *Polerovirus*	NC_000874	5,899	3467–3512	18 nt to CUG from ORF3a; 136 nt to AUG from ORF3–5
Beet Western yellows virus	BWYV	*Luteoviridae*; *Polerovirus*	NC_004756	5,666	3346–3393	Defective ORF3a; 138 nt to AUG from ORF3–5
Beet Western yellows luteovirus (strain bwyv-1, isolate 28a)	BWYV	*Luteoviridae*; *Polerovirus*	L39983	973	341–389	135 nt to AUG from ORF3–5
Hubei polero-like virus 2 (strain QTM26674)	HuPLV2	*Unclassified*	NC_033229	6,083	3706–3753	133 nt to AUG from ORF3–5
Hubei polero-like virus 1 (strain WHCC118254)	HuPLV1	*Unclassified*	NC_032224	4,213	3357–3410	135 nt to AUG from ORF3–5

a Viruses are grouped by the genomic context of the xrRNA (last column). The complete list of sequences used for comparative sequence alignment is shown in Table S1. Smirnova et al. (30) was used as a reference for updated annotations of ORF3a. BYDV, Barley yellow dwarf virus; nt, nucleotide(s).

b xrRNA boundaries are defined as the first nucleotide of the P1 stem and the last nucleotide of the pseudoknot.

10.1128/mBio.02461-18.3 FIG S3 Variable genomic location of xrRNA_D_ among plant viruses. (Left) Schematic genomic organization of plant virus genera that harbor xrRNA_D_ elements. xrRNA_D_ are depicted as red hexagons. Genomes are drawn to scale. (Right) sgRNAs or putative sgRNAs that would arise through incomplete degradation of the viral genome by 5´-to-3´ exoribonucleases. Note that the 5´ end of a sgRNA coincides with the 5´ end of xrRNA_D_ for *Dianthovirus* and *Machlomovirus*; the mapped 5´ ends of sgRNAs are slightly upstream of the xrRNA_D_ (indicated by dashed line) for *Polerovirus* and *Umbravirus*, suggesting that additional trimming events might be involved in sgRNA production. The asterisk indicates a second xrRNA_D_ element that was bioinformatically identified in OPMV but has not been tested biochemically. All other xrRNA_D_ elements depicted here have been biochemically verified *in vitro*. Download FIG S3, JPG file, 0.5 MB. Copyright © 2018 Steckelberg et al. 2018 Steckelberg et al. This content is distributed under the terms of the Creative Commons Attribution 4.0 International license.

The presence of xrRNA_D_ in intergenic regions and upstream of protein-coding sequences suggests several possibilities for the role of xrRNAs in this new context. First, these intergenic xrRNA_D_ could be used to produce sgRNAs similarly to the role ascribed to xrRNAs in 3′UTRs; that is, sgRNAs could be produced by incomplete degradation of full-length genomic RNAs without requiring a subgenomic promoter. Alternatively, precursor sgRNAs could be produced by transcription from a subgenomic promoter or from templates made by premature termination during negative-strand synthesis. These precursor RNAs could be subsequently “trimmed” by exonucleases to yield a mature sgRNA. In this scenario, the transcription start site could be at any distance from the 5′ end of the mature sgRNA. Another possibility is that the sgRNA could be produced by transcription from a subgenomic promoter and that the role of the xrRNA would be to protect sgRNA from 5′-to-3′ degradation by cellular exoribonucleases. Our data suggest that all of the scenarios are possible and that the roles of intergenic xrRNA_D_ may be different in different viruses. For example, unlike in MCMV, the 5′ end of the xrRNA_D_ sequence in PLRV (*Luteoviridae*; *Polerovirus*) does not correspond to the mapped 5′ end of the sgRNA1. Rather, it is located 28 nucleotides upstream of the proposed exoribonuclease stop site. Furthermore, previous studies showed that PLRV sgRNA1 is likely generated by a replicative mechanism and thus that the xrRNA in PLRV probably does not directly function in the initial generation of sgRNA1 (33). This organization is also found in beet Western yellows virus (also a polerovirus) (34). In this case, the xrRNA_D_ might be involved in a regulatory “trimming” step that alters the 5′ end of existing transcripts, a process that may therefore occur only under certain conditions or at certain times in viral infection. Before testing this hypothesis, it would be crucial to determine if, when, and where xrRNA-dependent sgRNAs accumulate in infected cells and if there is variation in the 5′ end within the population of a specific sgRNA. Moreover, mapping precise 5′ ends of additional sgRNAs from different virus species (and comparing them to the location of xrRNA_D_) is needed to provide insight into the potential functions of xrRNAs during the generation, maintenance, and regulation of viral coding and noncoding sgRNAs.

That xrRNA_D_ are at or near the 5′ end of protein coding sgRNAs raises the issue of whether or not sgRNAs produced or maintained by intergenic xrRNA_D_ can be translated. The 5′ end of sgRNAs resulting from xrRNA-dependent halting of 5′-to-3′ degradation would not have a modified nucleotide 5′ “cap”; thus, sgRNAs lack the canonical translation initiation signal. However, since viruses of the *Tombusviridae* family use 3′-proximal cap-independent translation enhancers (3′-CITEs) to initiate translation, uncapped sgRNAs with xrRNAs on their 5′ ends could potentially be translationally active (35, 36). In addition, it has been shown that some of these viruses use diverse 3′-CITEs and different amounts of various sgRNA species to fine-tune viral protein production during infection. Thus, these xrRNAs could be part of a larger RNA structure-dependent mechanism involving 3′-CITEs and xrRNAs in regulation of both the amount and translational activity of protein-encoding viral genomic RNAs and sgRNAs (37). Again, the details of such mechanisms would almost certainly differ in various viral species.

In contrast to the members of the *Tombusviridae* family, not all *Luteoviridae* members contain 3′CITEs; thus, if xrRNA_D_-associated sgRNAs are translated, they must use a different mechanism of translation initiation. For example, in the *Polerovirus* genus, cap-independent translation is likely conferred by genome-linked proteins (VPg) that are covalently attached to the 5′ end of the viral genome (W. Allen Miller, personal communication). The related sobemoviruses also have a VPg attached to their sgRNAs (38–41), but whether this is also true of polerovirus sgRNAs is currently unknown. Any sgRNAs resulting from xrRNA_D_-dependent exoribonuclease resistance would be expected to have a 5′ monophosphate and not a VPg, raising the possibilities that such sgRNAs would be translationally inactive but could be maintained for some regulatory purpose and that there could be pools of translationally active and inactive sgRNAs. These possibilities all remain speculative; understanding the purpose of xrRNA_D_ in each virus, how they relate to translation, and the existence of any larger trends will require ongoing detailed studies of diverse viruses.

Overall, our discoveries suggest that the roles of xrRNAs are more diverse than previously realized, depending on their genetic context. The presence of xrRNAs in various locations within viral genomes suggests that new xrRNA scaffolds may emerge from analyzing sgRNA 5′ termini from other viruses; certainly, not all xrRNA elements were identified by the algorithm used here (5, 7, 42). Intriguing candidates for novel xrRNA identification include viruses with no obvious upstream promoter elements for sgRNA production and viruses in which putative promoter sequences do not seem to correspond well to the sgRNA 5′ end (1, 5, 42, 43).

Many issues remain that pertain to understanding the structural/sequence requirements for Xrn1 resistance, the degree to which structural variation is tolerated, and how sequence diversity is integrated into similar folds (44). The now-expanded set of xrRNA_D_ candidates provides a broader phylogeny for future bioinformatic and structural studies that will address these points.

## MATERIALS AND METHODS

### Computational search.

The published alignment performed with sequences from a total of three virus species (RCNMV, SCNMV, and CRSV) (26) was manually expanded in Ugene v. 1.29.0 (45) with two RCNMV variants (GenBank accession no. J04357 and AB034916) retrieved from a standard nucleotide BLAST search for “somewhat dissimilar sequences” (https://blast.ncbi.nlm.nih.gov/Blast.cgi?PAGE_TYPE=BlastSearch). Sequences were aligned to the conserved 3D-based secondary structure, omitting the pseudoknot, and were exported in Stockholm format (see Fig. S4A in the supplemental material).

10.1128/mBio.02461-18.3 FIG S4 Alignments. Download FIG S4, PDF file, 0.3 MB. Copyright © 2018 Steckelberg et al. 2018 Steckelberg et al. This content is distributed under the terms of the Creative Commons Attribution 4.0 International license.

Using *Infernal* v. 1.1.2 (27) with default parameters, we searched for domains with similar structures and sequences within the complete reference genomes of viruses available from RefSeq, the NCBI Reference Sequence Database (https://www.ncbi.nlm.nih.gov/refseq/; downloaded on 10 January 2018). For subsequent iterations with *Infernal*, we searched the complete database of *Tombusviridae* and *Luteoviridae* available at GenBank (downloaded on 3 July 2018), using the alignment shown in Fig. S4B in the supplemental material.

In Ugene, we systematically added new hits from *Infernal* to the alignment only when they met the following criteria: (i) the sequence showed variation in more than 3 to 5 locations from the sequences already in the alignment; (ii) the *Infernal* E value was <0.05; (iii) the *Infernal* score was >10; (iv) the genomic context was coherent with that of the sequences already in the alignment. But a key objective in expanding the alignment further was also to analyze potential hits with a higher E value/a lower score, as they would often correspond to positive hits but with a larger sequence or structure variation. By the time the alignment reached a size of 10 to 12 sequences, we were able to retrieve most of the sequences that made it into the final alignment through further iterations of *Infernal* searches and manual addition to the alignment. Hits for unclassified viruses were also retrieved from large-scale transcriptomics data of invertebrate and vertebrate-associated RNA viruses using the deposited sequences (46, 47).

A statistical validation of the final proposed alignment of 47 sequences was performed using the latest version of R-scape available at http://eddylab.org/R-scape/ (29) (last accessed on 17 August 2018). The corresponding conserved structure and sequence patterns were rendered using R2R v. 1.0.5 (48).

### Design of RNAs for *in vitro* assays.

The DNA templates used for *in vitro* transcription were gBlocks ordered from IDT and were cloned into pUC19 and verified by sequencing. RNA constructs for Xrn1 degradation assays contained the xrRNA sequence plus ∼30 nucleotides of the endogenous upstream sequence (“leader sequence”) to allow loading of the exoribonucleases. Table 2 shows the sequences used in *in vitro* Xrn1 degradation assays.

**TABLE 2:** Sequences used in the *in vitro* Xrn1 degradation assays^*a*^

RNA	Sequence (5′–3′)
OPMV xrRNA	TAATACGACTCACTATA*GGAATTGCCTCCACCAGTAACTAAACCCA****A*C**CACAGCCAAGCATTAAGTTGCAAGCGTTGGAGTGGCAGGCTT AACGTCCGACAGTACGACAACTGCGG
MCMV xrRNA	TAATACGACTCACTATA*GGTTCCAGGCCCAGGGCTGGCAAATCATTGAGCACAAG***G**TGAGCCGGCATGAGGTTGCAAGACCGGAACAACC AGTCCTTCTGGCAGAGTCCTGCCAA
PLRV xrRNA	TAATACGACTCACTATAg*GCCACCACAAAAGAACACTGAAGGAGCTCACTA*AAACTAGCCAAGCATACACGAGTTGCAAGCATTGGAAGT TCAAGCCTCGT
MYDV-RMV xrRNA	TAATACGACTCACTATAg*GTCCAGAAACAAAAAGTTTAAAACAGA*A*GCTCTCA*AGTCAGCCAGGCAAATTCGAGTTGCAAGCACTGGATG ACCTAGTCTCGATA
HuPLV1 xrRNA	TAATACGACTCACTATAg*GCCACAAAACGAATAAAGGAAGAACGCACGA*GAGTCAGCCAAACAAACACAAGTTGCAAGTGTTGGAGACT CATTCTAGTCTTGT

a The T7 promoter sequences are underlined, the leader sequence are indicated in italics, and the first protected nucleotides (experimentally validated as described here) are indicated in bold. Lowercase letters indicate extra nucleotides inserted to allow better transcription.

### RNA preparation.

DNA templates for *in vitro* transcription were amplified by PCR using custom DNA primers (IDT) and Phusion Hot Start polymerase (New England BioLabs). Transcription reaction mixtures (2.5 ml) were assembled using 1,000-µl PCR reaction mixtures as the template (∼0.2 µM template DNA, a 6 mM concentration of each NTP, 60 mM MgCl_2_, 30 mM Tris [pH 8.0], 10 mM dithiothreitol [DTT], 0.1% spermidine, 0.1% Triton X-100, T7 RNA polymerase, and 2 µl RNasin RNase inhibitor [Promega]) and incubated overnight at 37°C. After inorganic pyrophosphates were precipitated by centrifugation, the reaction mixtures were ethanol precipitated and purified on a 7 M urea–8% denaturing polyacrylamide gel. RNAs of the correct size were excised, eluted overnight at 4°C into ∼40 ml of diethylpyrocarbonate (DEPC)-treated Milli-Q filtered water (Millipore), and concentrated using Amicon Ultra spin concentrators (Millipore). Mutations were introduced using mutagenized custom DNA reverse primers (Table 3).

**TABLE 3:** Primers used in this study^*a*^

Primer	Sequence (5′–3′)
OPMV_WT_rev	5′-CCGCAGTTGTCGTACTGTCGG-3′
OPMV_PKmut1_rev	5′-CCGCAGTTGTCGTACTGTCGGACG**AATT**GCCTGCCACTCCAACGC-3′
OPMV_PKmut2_rev	5′-CCGCAGTTGTCGTACTGTCGGACGTTAAGCCTGCCACTCCAACGCTTGCAAC**AATT**TGCTT GGCTGTGGTTGG-3′
OPMV_PKcomp_rev	5′-CCGCAGTTGTCGTACTGTCGGACG**AATT**GCCTGCCACTCCAACGCTTGCAAC**AATT**TGCTT GGCT GTGGTTGG-3′
MCMV_WT_rev	5′-TGGCAGGACTCTGCCAGAAGG-3′
MCMV_PKmut1_rev	5′-TGGCAGGACTCTGCCAG**CTCC**ACTGGTTGTTCCGGTCTTGC-3′
MCMV_PKmut2_rev	5′-TGGCAGGACTCTGCCAGAAGGACTGGTTGTTCCGGTCTTGCAA**GGAG**ATGCCGGCTCACC TTGTGCTC-3′
MCMV_PKcomp_rev	5′-TGGCAGGACTCTGCCAG**CTCC**ACTGGTTGTTCCGGTCTTGCAA**GGAG**ATGCCGGCTCACC TTGTGCTC-3′
PLRV_WT_rev	5′-ACGAGGCTTGAACTTCCAATGC-3′
PLRV_PKmut1_rev	5′-**TGCT**GGCTTGAACTTCCAATGCTTGC-3′
PLRV_PKmut2_rev	5′-ACGAGGCTTGAACTTCCAATGCTTGCAAC**AGCA**GTATGCTTGGCTAGTTTTAGTG-3′
PLRV_PKcomp_rev	5′-**TGCT**GGCTTGAACTTCCAATGCTTGCAAC**AGCA**GTATGCTTGGCTAGTTTTAGTG-3′
MYDV-RMV_WT_rev	5′-TATCGAGACTAGGTCATCCAGTGC-3′
huPLV_WT_rev	5′-ACAAGACTAGAATGAGTCTCC-3′
huPLV_PKmut1_rev	5′-**TGTT**GACTAGAATGAGTCTCCAACACTTGC-3′
huPLV_PKmut2_rev	5′-ACAAGACTAGAATGAGTCTCCAACACTTGCAAC**AACA**GTTTGTTTGGCTGACTCTCG-3′
huPLV_PKcomp_rev	5′-**TGTT**GACTAGAATGAGTCTCCAACACTTGCAAC**AACA**GTTTGTTTGGCTGACTCTCG-3′

a Mutated residues are indicated in bold.

### *In vitro* Xrn1 resistance assays.

RNA (4 µg) was resuspended in 40 µl 100 mM NaCl–10 mM MgCl_2_–50 mM Tris (pH 7.5)–1 mM DTT and refolded at 90°C for 3 min and then at 20°C for 5 min. A 3-µl volume of recombinant RppH (0.5 µg/µl stock) was added, and the samples were split into two 20-µl reaction mixtures (with or without exoribonuclease). A 1-µl volume of the recombinant Xrn1 (0.8 µg/µl stock) was added where indicated. All reaction mixtures were incubated for 2 h at 30°C using a thermocycler. The degradation reaction mixtures were resolved on a 7 M urea–8% denaturing polyacrylamide gel and stained with ethidium bromide.

### Mapping of the exoribonuclease stop site.

To determine the Xrn1 stop site at single-nucleotide resolution, 30 µg *in vitro*-transcribed RNA was degraded using recombinant RppH and Xrn1 as described above (the reaction volume was scaled up to 300 µl, and 20 µl of each enzyme was used). The degradation reaction mixture was resolved on a 7 M urea–8% polyacrylamide gel, and then the Xrn1-resistant degradation product was cut from the gel and eluted overnight at 4°C into ∼20 ml of diethylpyrocarbonate (DEPC)-treated Milli-Q filtered water (Millipore) and concentrated using Amicon Ultra spin concentrators (Millipore). Once recovered, the RNA was subjected to reverse transcription (RT) using Superscript III reverse transcriptase (Thermo) and a 6-carboxyfluorescein (FAM) (6-fluorescein amidite)-labeled sequence-specific reverse primer (IDT) with a 20 (A) stretch at the 5′ end to allow cDNA purification with oligo(dT) beads. The RT reaction volumes (5 µl) contained 1.2 pM RNA, 0.25 µl 0.25 µM FAM-labeled reverse primer, 1 µl 5× first-strand buffer, 0.25 µl 0.1 M DTT, 0.4 µl 10 mM deoxynucleoside triphosphate (dNTP) mix, and 0.1 µl Superscript III reverse transcriptase (200 U/µl) and were incubated for 1 h at 50°C. To hydrolyze the RNA template after reverse transcription, 5 µl of 0.4 M NaOH was added and the reaction mixture incubated at 90°C for 3 min, followed by cooling on ice for 3 min. The reaction was neutralized by adding 5 µl of acid quench mix (1.4 M NaCl, 0.57 M HCl, 1.3 M sodium acetate, pH 5.2), and then 1.5 µl of oligo(dT) beads [Poly(A)Purist MAG kit (Thermo)] was added and the cDNA was purified on a magnetic stand according to the manufacturer’s instructions. The cDNA was eluted in 11 µl ROX-HiDi and analyzed on a model 3500 Genetic Analyzer (Applied Biosystems) for capillary electrophoresis. A Sanger sequencing (ddNTP) ladder of the undigested RNA was analyzed alongside each degradation product as a reference for band annotation.

### Data availability.

All data are available from us.
