# Supplementary figures and images for: Exoribonuclease-Resistant RNAs Exist within both Coding and Noncoding Subgenomic RNAs
Source: mBio. 2018 Dec 18;9(6):e02461-18. doi: 10.1128/mBio.02461-18 (PMC6299227; doi:10.1128/mBio.02461-18)

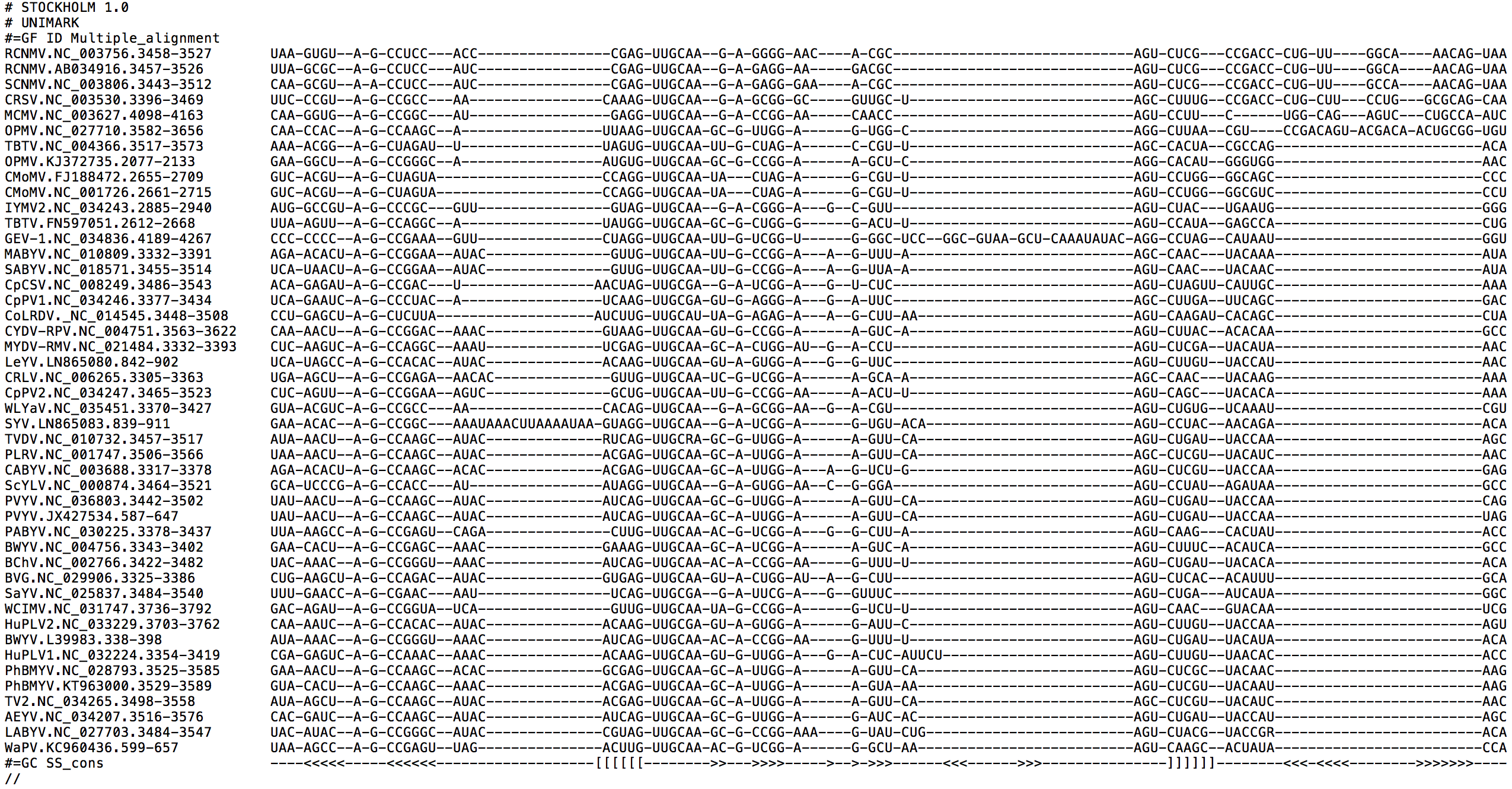

Supplement: FIG S1 [file mbo006184225sf1.tif]

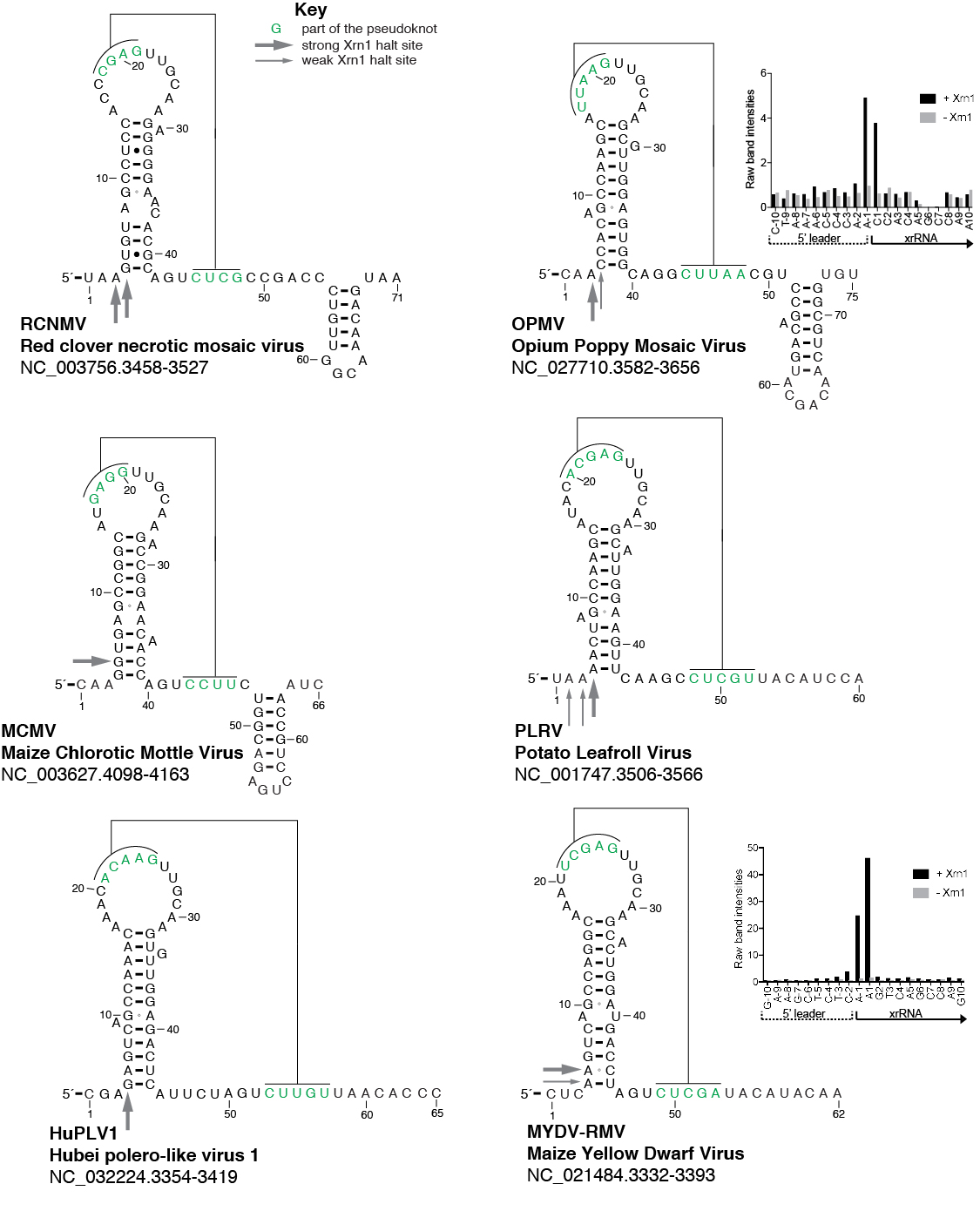

Supplement: FIG S2 [file mbo006184225sf2.jpg]

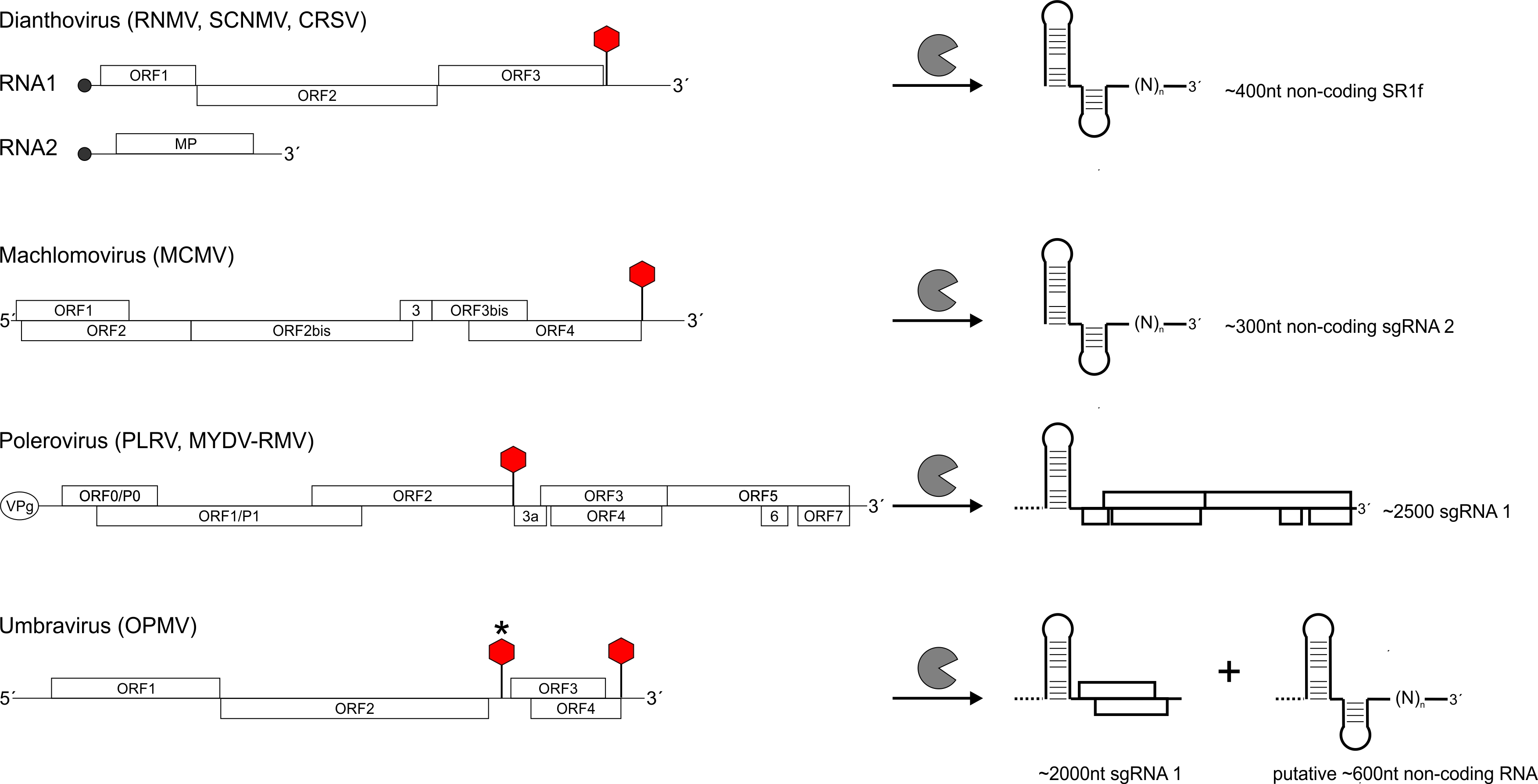

Supplement: FIG S3 [file mbo006184225sf3.jpg]
