# Supplementary material for: Exoribonuclease-Resistant RNAs Exist within both Coding and Noncoding Subgenomic RNAs
Source: mBio. 2018 Dec 18;9(6):e02461-18. doi: 10.1128/mBio.02461-18 (PMC6299227; doi:10.1128/mBio.02461-18)
Supplement: TABLE S1 [file mbo006184225st1.pdf]

Abbreviation	Classification	GenBank ID	Total ssRNA length (nt)	xrRNA boundaries (from the 1st nucleotide of the P1 stem to the last nucleotide of the pseudoknot)	Sequence range shown in sequence alignment (i.e., comprising xrRNA flanking sequences)	Genomic context (for 3'UTR xrRNA, distance up to the first nucleotide of the P1 stem; for intergenic xrRNAs, distance from the 3'-most nucleotide of the pseudoknot)
RCNMV	Tombusviridae; Dianthovirus	NC_003756	3890	3461-3504	3458-3527	3' UTR; 11 nt downstream from STOP codon
RCNMV	Tombusviridae; Dianthovirus	AB034916	3890	3460-3503	3457-3526	3' UTR; 11 nt from STOP codon
SCNMV	Tombusviridae; Dianthovirus	NC_003806	3876	3446-3489	3443-3512	3' UTR; 3 nt from STOP codon
CRSV	Tombusviridae; Dianthovirus	NC_003530	3840	3399-3444	3396-3469	Frameshifting element close to 3' UTR
MCMV	Tombusviridae; Machlomovirus	NC_003627	4437	4101-4143	4098-4163	3' UTR; coincides with beginning of sgRNA2
OPMV	Tombusviridae; Umbravirus	NC_027710	4230	3585-3629	3582-3656	3' UTR; 4 nt from STOP codon
TBTV	Tombusviridae; Umbravirus	NC_004366	4152	3520-3564	3517-3573	3' UTR; 10 nt from STOP codon
OPMV	Tombusviridae; Umbravirus	KJ372735	2739	2080-2124	2077-2133	69 nt to AUG from ORF3 (starts at 2193)
CMoMV	Tombusviridae; Umbravirus	FJ188472	3957	2658-2700	2655-2709	73 nt to AUG from "long-distance movement protein" (starts at 2773)
CMoMV	Tombusviridae; Umbravirus	NC_001726	4201	2664-2706	2661-2715	74 nt to AUG from ORF3 (starts at 2790)
IYMV2	Tombusviridae; Umbravirus	NC_034243	4196	2888-2931	2885-2940	77 nt to AUG from ORF3 (starts at 3008)
TBTV	Tombusviridae; Umbravirus	FN597051	4152	2615-2659	2612-2668	98 nt to AUG from ORF3 (starts at 2757)
GEV-1	Luteoviridae; Enamovirus; unclassified	NC_034836	6176	4192-4259	4189-4267	5 nt to AUG from ORF3 (starts at 4264)
MABYV	Luteoviridae; Polerovirus	NC_010809	5674	3335-3382	3332-3391	7 nt to AUA from ORF3a (starts at 3389); 123 nt to AUG from ORF3-5 (starts at 3516)
SABYV	Luteoviridae; Polerovirus	NC_018571	5843	3458-3505	3455-3514	7 nt to AUA from ORF3a (starts at 3512); 134 nt to AUG from ORF3-5 (starts at 3639)
CpCSV	Luteoviridae; Polerovirus	NC_008249	5900	3489-3534	3486-3543	11 nt to AUA from ORF3a (starts at 3545); 129 nt to AUG from ORF3-5 (starts at 3663)
CpPV1	Luteoviridae; Polerovirus	NC_034246	5845	3380-3425	3377-3434	11 nt to CUG from ORF3a (starts at 3436); 129 nt to AUG from ORF3-5 (starts at 3554)
CoLRDV	Luteoviridae; Polerovirus	NC_014545	5866	3451-3499	3448-3508	13 nt to CUG from ORF3a (starts at 3512); 131 nt to AUG from ORF3-5 (starts at 3630)
CYDV-RPV	Luteoviridae; Polerovirus	NC_004751	5723	3566-3622	3563-3613	14 nt to AUU from ORF3a (starts at 3627); 132 nt to AUG from ORF3-5 (starts at 3745)
MYDV-RMV	Luteoviridae; Polerovirus	NC_021484	5612	3335-3384	3332-3393	14 nt to ACG from ORF3a (starts at 3398); 132 nt to AUG from ORF3-5 (starts at 3516)
LeYV	Luteoviridae; Polerovirus	LN865080	1625	845-893	842-902	15 nt to AUA from ORF3 (starts at 908); 133 nt to AUG from ORF3-5 (starts at 1026)
CRLV	Luteoviridae; Polerovirus	NC_006265	5723	3308-3354	3305-3363	16 nt to AUA from ORF3a (starts at 3370); 157 nt to AUG from ORF3-5 (starts at 3511)
CpPV2	Luteoviridae; Polerovirus	NC_034247	5945	3468-3514	3465-3523	16 to AUA from ORF3a (starts at 3530); 134 nt to AUG from ORF3-5 (starts at 3648)
WLYaV	Luteoviridae; Polerovirus	NC_035451	5772	3373-3418	3370-3427	17 nt to CUG from ORF3a (starts at 3435); 135 nt to AUG from ORF3-5 (starts at 3553)
SYV	Luteoviridae; Polerovirus	LN865083	1636	842-902	839-911	17 nt to AUA from ORF3a (starts at 919); 135 nt to AUG from ORF3-5 (starts at 1037)
TVDV	Luteoviridae; Polerovirus	NC_010732	5920	3460-3508	3457-3517	18 nt to AUA from ORF3a (starts at 3526); 136 nt to AUG from ORF3-5 (starts at 3644)
PLRV	Luteoviridae; Polerovirus	NC_001747	5987	3509-3557	3506-3566	18 nt to AUA from ORF3a (starts at 3575); 136 nt to AUG from ORF3-5 (starts at 3693)
CABYV	Luteoviridae; Polerovirus	NC_003688	5669	3320-3369	3317-3378	18 nt to AUA from ORF3a (starts at 3387); 136 nt to AUG from ORF3-5 (starts at 3505)
ScYLV	Luteoviridae; Polerovirus	NC_000874	5899	3467-3512	3464-3521	18 nt to CUG from ORF3a (starts at 3530); 136 nt to AUG from ORF3-5 (starts at 3648)
PVYV	Luteoviridae; Polerovirus	NC_036803	6125	3445-3493	3442-3502	19 nt to AUA from ORF3 (starts at 3512); 137 nt to AUG from ORF3-5 (starts at 3630)
PVYV	Luteoviridae; Polerovirus	JX427534	1381	590-638	587-647	19 nt to AUA from ORF3a (starts at 657); 137 nt to AUG from ORF3-5 (starts at 775)
PABYV	Luteoviridae; Polerovirus	NC_030225	5813	3381-3428	3378-3437	22 nt to ACG from ORF3a (starts at 3450); 140 nt to AUG from ORF3-5 (starts at 3568)
BWYV	Luteoviridae; Polerovirus	NC_004756	5666	3346-3393	3343-3402	ORF3a defective; 138 nt to AUG from ORF3-5 (starts at 3531)
BChV	Luteoviridae; Polerovirus	NC_002766	5776	3425-3473	3422-3482	ORF3a defective; 134 nt to AUG from ORF3-5 (starts at 3607)
BVG	Luteoviridae; Polerovirus	NC_029906	5620	3328-3377	3325-3386	131 nt to AUG from ORF3-5 (starts at 3508)
SaYV	Luteoviridae; Polerovirus	NC_025837	5820	3487-3531	3484-3540	131 nt to AUG from ORF3-5 (starts at 3662)
WClMV	Luteoviridae; Polerovirus	NC_031747	6205	3739-3783	3736-3792	133 nt to AUG from ORF3-5 (starts at 3916)
HuPLV2	Picornaviridae unclassified	NC_033229	6083	3706-3753	3703-3762	133 nt to AUG from ORF3-5 (starts at 3886)
BWYV	Luteoviridae; Polerovirus	L39983	973	341-389	338-398	135 nt to AUG from ORF3-5 (starts at 524)
HuPLV1	Picornaviridae unclassified	NC_032224	4213	3357-3410	3354-3419	135 nt to AUG from ORF3-5 (starts at 3545)
PhBMYV	Luteoviridae; Polerovirus	NC_028793	5867	3528-3576	3525-3585	135 nt to AUG from ORF3-5 (starts at 3711)
PhBMYV	Luteoviridae; Polerovirus	KT963000	5855	3532-3580	3529-3589	135 nt to AUG from ORF3-5 (starts at 3715)
TV2	Luteoviridae; Polerovirus	NC_034265	5979	3501-3549	3498-3558	136 nt to AUG from ORF3-5 (starts at 3685)
AEYV	Luteoviridae; Polerovirus	NC_034207	5953	3519-3567	3516-3576	137 nt to AUG from ORF3-5 (starts at 3704)
LABYV	Luteoviridae; Polerovirus	NC_027703	5961	3487-3538	3484-3547	139 nt to AUG from ORF3-5 (starts at 3677)
WaPV	Luteoviridae; Polerovirus	KC960436	1025	602-649	599-657	139 nt to AUG from ORF3-5 (starts at 788)
