# Supplementary material for: Exoribonuclease-Resistant RNAs Exist within both Coding and Noncoding Subgenomic RNAs
Source: mBio. 2018 Dec 18;9(6):e02461-18. doi: 10.1128/mBio.02461-18 (PMC6299227; doi:10.1128/mBio.02461-18)
Supplement: FIG S4 [file mbo006184225sf4.pdf]

A

```
# STOCKHOLM 1.0
# UNIMARK
#=GF ID Multiple_alignment
RedCloverNecroticMosaicVirus_1              GCGUAGCCUCCACCCGAGUUGCAAGAG-GGAACGCGC-AGUCUCG-CC
RedCloverNecroticMosaicVirus_J04357-1       GCGUAGCCUCCACCCGAGUUGCAAGAGGGGAACACGC-AGUCUCG-CC
RedCloverNecroticMosaicVirus_AB034916-1     GCGCAGCCUCCAUCCGAGUUGCAAGAGAGGAAGACGC-AGUCUCG-CC
SweetCloverNecroticMosaicVirus_L07884-1     GCGUAACCUCCAUCCGAGUUGCAAGAGAGGGAAACGC-AGUCUCG-CC
CarnationRingSpotVirus_L18870-2             CCGUAGCCGCCAACAAAGUUGCAAGAGCGGGCGUUGCUAGCCUUUGCC
#=GC SS_cons                                <<<<--<<<<<-------------->->>>>--->>>>-----------
//
```

B

```
# STOCKHOLM 1.0
# UNIMARK
#=GF ID Multiple_alignment
RedCloverNecroticMosaicVirus_1                 -GCGUAGCCUCCACC-CGAG-UUGCAAGAG-GGAACGCGC-AGU-CUCG--CC
RedCloverNecroticMosaicVirus_J04357-1          -GCGUAGCCUCCACC-CGAG-UUGCAAGAGGGGAACACGC-AGU-CUCG--CC
RedCloverNecroticMosaicVirus_AB034916-1        -GCGCAGCCUCCAUC-CGAG-UUGCAAGAGAGGAAGACGC-AGU-CUCG--CC
SweetCloverNecroticMosaicVirus_L07884-1        -GCGUAACCUCCAUC-CGAG-UUGCAAGAGAGGGAAACGC-AGU-CUCG--CC
CarnationRingSpotVirus_L18870-2                -CCGUAGCCGCCAAC-AAAG-UUGCAAGAGCGGGCGUUGCUAGC-CUUU-GCC
WheatLeafYellowingAssociatedVirus_NC_035451  ACGUCAGCCGCCAAC-ACAG-UUGCAAGAGCGGAAGACG-UAGU-CUGU-GUC
SugarcaneYellowLeafVirus_NC_000874             CCCGAGCCACCAUA-UAGG-UUGCAAGAGUGGAACGGGA-AGU-CCUA-UA-
#=GC SS_cons                                   -<<<<-<<<<<<----------<--->>->>>>>-->>>>-------------
//
```
